# Synergistic glycolysis disturbance for cancer therapy by a MOF-based nanospoiler

**DOI:** 10.52601/bpr.2023.230003

**Published:** 2023-06-30

**Authors:** Xuemei Zeng, Yihang Ruan, Lun Wang, Jinpeng Deng, Shuangqian Yan

**Affiliations:** 1 Key Laboratory of Innate Immune Biology of Fujian Province, Biomedical Research Center of South China, College of Life Sciences, Fujian Normal University, Fuzhou 350117, China; 2 The Straits Institute of Flexible Electronics (SIFE, Future Technologies), the Straits Laboratory of Flexible Electronics (SLoFE), Fujian Normal University, Fuzhou 350117, China

**Keywords:** Glycolysis disturbance, Energy deprivation, Drug delivery, Zeolitic imidazolate frameworks, Metal-organic frameworks

## Abstract

Increased glycolysis for promoting adenosine triphosphate (ATP) generation is one of the hallmarks of cancer. Although reducing glucose intake or depriving cellular glucose can delay the growth of tumors to some extent, their therapeutic efficacy is a highly needed improvement for clinical translation. Herein, we found that mannose synergistic with glucose oxidase (GOx) can induce cell death by ATP inhibition, autophagy activation, and apoptosis protein upgradation. By using biodegradable zeolitic imidazolate frameworks (ZIF-8) as a nanocarrier (denoted as ZIF-8/M&G), the mannose and GOx can accumulate at the tumor site while having no obvious long-term toxicity. At the tumor site, GOx inhibits glycolysis by converting glucose and oxygen to H _2_O _2_ and gluconic acid, realizing oxidation therapy and expediting the degradation of the pH-responsive ZIF-8 nanoparticles, respectively. Simultaneously, mannose disturbs sugar metabolism and reduces oxygen consumption, which in turn promotes the GOx oxidation process. The concerted glycolysis inhibition through interactions between mannose and GOx endows ZIF-8/M&G nanospolier with excellent therapeutic efficacy both *in vitro* and *in vivo*. Synergistic glycolysis disturbance by the designed nanospoiler in this work proposes a versatile approach for metabolism disturbance to tumor treatment.

## INTRODUCTION

Cancer cells possess abnormal metabolism to meet the demands for infinite proliferation (DeBerardinis 2008; DeBerardinis and Chandel 2016; Seyfried and Shelton 2010). Due to mitochondrial defects, hypoxia, and altered metabolic enzymes, the metabolism in cancer cells switches from oxidative phosphorylation to extensive glycolysis even if the oxygen is sufficient (the Warburg effect) (Liberti and Locasale 2016). Most cancer cells largely depended on glycolysis to generate adenosine triphosphate (ATP) for proliferation and survival (Oronsky *et al.* 2014). Thus, inhibition of glycolysis is a promising strategy for tumor treatment by metabolism disturbance and energy deprivation (Ganapathy-Kanniappan and Geschwind 2013; Porporato *et al.* 2011; Zhao *et al.* 2013).

There is an emerging variety of methods for glycolysis modulation with a deeper understanding of the molecular mechanisms of cancer cells (Pelicano *et al.* 2006; Xu *et al.* 2005). And a diverse set of inhibitors and compounds were utilized to modulate glycolytic metabolism in different manners. It was worth noting that glucose, the source of cellular energy, can be controlled by fasting or fasting-mimicking diets and direct consumption (Brandhorst *et al.* 2015; Di Biase *et al.* 2016). In principle, cancer cells increase the glucose uptake to facilitate a higher rate of glycolysis (Gillies *et al.* 2008; Nencioni *et al.* 2018; Pavlova and Thompson 2016; Shaw 2006). Correspondingly, the high concentration of cellular glucose is not only a biomarker for tumor cancerization but a target spot for tumor therapy (Hay 2016). For instance, glucose oxidase (GOx) and gold nanoparticles with GOx-mimicking properties are widely used to deprive glucose in cancer cells, realizing starvation and oxidation tumor therapy. However, the therapeutic effects of GOx or GOx mimics are limited due to the hypoxia of the tumor microenvironment. Although other therapeutic manners such as phototherapy and chemical therapy can improve the efficacy of the GOx-based tumor treatment, it is challenging to inhibit glycolysis for tumor therapy with high efficiency (Fu *et al.* 2018, 2019; Zeng *et al.* 2022). Mannose, a glucose analog, used by the same transporters of glucose (Thorens and Mueckler 2010), can be converted to mannose-6-phosphate in cells thus restraining enzymatic activities of glucose metabolism and inhibiting glycolysis. Its antitumor activity has been verified in mouse models recently (Fan *et al.* 2020; Gonzalez *et al.* 2018). However, mannose can increase intracellular glucose, and a high concentration of phosphomannose isomerase counteracts glycolysis inhibition efficiency. Thus, we think mannose and GOx may enhance each other for tumor treatment through high-efficient glycolysis inhibition.

In this work, we designed a nanospoiler biomineralization from zeolitic imidazolate framework nanoparticle (ZIF-8) mannose, and GOx (denoted as ZIF-8/M&G) to synergistic glycolysis disturbance for tumor therapy ( Scheme 1) (Chen *et al.* 2018; Liang *et al.* 2015; Yan *et al.* 2020). The reasons for the nanospoiler design are as follows: (1) ZIF-8 has properties of good biocompatibility, easy fabrication, and pH-responsive biodegradability, which is an intelligent nanocarrier for therapeutic delivery (Qian *et al.* 2022; Sun *et al.* 2012, 2019); (2) mannose interferes with glucose metabolism thus elevating the oxygen content, which is favorable to GOx oxidation (Gonzalez *et al.* 2018); (3) GOx transfers glucose and oxygen to gluconic acid and H _2_O _2_, realizing oxidation therapy and tumor starvation (Fan *et al.* 2017); and (4) the generated gluconic acid, in turn, favors the degradation of ZIF-8/M&G to enhance the release of GOx and mannose (Cheng *et al.* 2019). Collectively, the as-prepared nanospoiler shows promise in tumor treatment by disturbing glucose metabolism.

**Figure 1:**
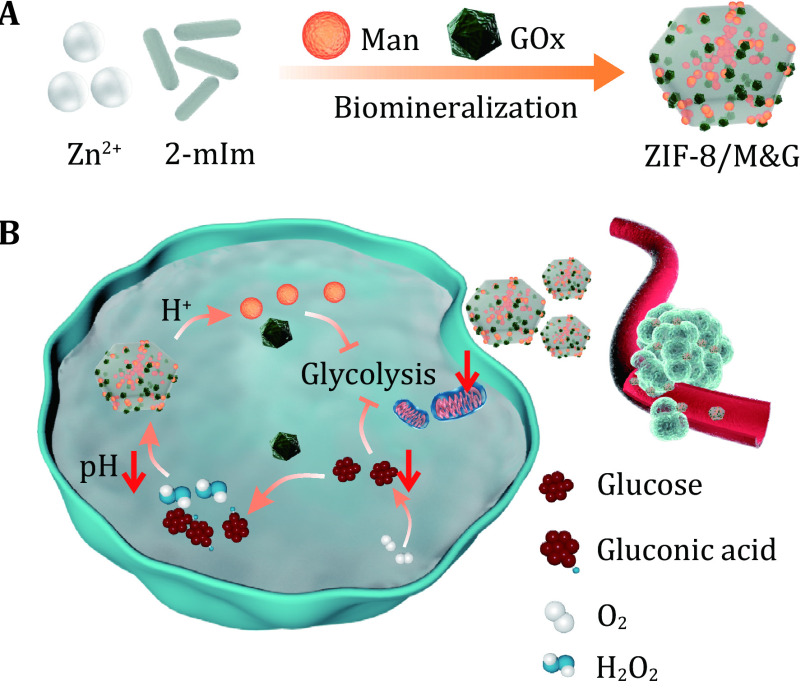
Schematic presentation of ZIF-8/M&G fabrication ( **A**) and tumor therapy by synergistic glycolysis inhibition ( **B**)

## RESULTS AND DISCUSSION

### Evaluation of the synergistic therapeutic efficacy of free mannose plus GOx *in vitro*

Glucose and mannose share the same transporter, GLUT1, to enter cells, which are metabolized into glucose-6-phosphate and mannose-6-phosphate by hexokinases (HK), respectively ( Fig. 1A). The generated glucose-6-phosphate further metabolizes into fructose-6-phosphate by phosphoglucose isomerase (PGI) while mannose-6-phosphate transforms to fructose-6-phosphate through phosphomannose isomerase (PMI) (DeRossi *et al.* 2006). Significantly, the accumulation of mannose-6-phosphate will disturb glucose metabolism by inhibiting multiple enzymes including HK, glucose-6-phosphate dehydrogenase (G6PD), and PGI. Furthermore, the sluggish glucose metabolism induced by mannose will relieve oxygen consumption, which may benefit the catalytic effect of the GOx. Thus, we think that the combination of mannose and GOx may treat tumors through concerted glycolysis inhibition. To validate our hypothesis, we conducted cell viability and apoptosis experiments *in vitro*. Firstly, we incubated mannose (5 mg/mL) with NIH 3T3, breast cancer cell line MCF-7, and lung cancer cell line A549 cells. According to cellular apoptosis analysis ( Fig. 1B), mannose induced minimal apoptosis in 3T3 and MCF-7 cells while having a high apoptosis rate in A549 cells. This result confirmed that mannose has the ability to induce apoptosis in the cancer cell with low PMI expression (that is a metabolizing enzyme for mannose glycolysis). Then, A549 cells were incubated with free mannose and GOx with various ratios. As shown in Fig. 1C, mannose has minimal effects on cell viability while GOx inhibited cell viability significantly, which arises from the high glucose consumption efficiency of GOx. Interestingly, cell treatment with mannose and GOx has lower viability than mannose or GOx treatment, showing the potential concerted effects of mannose and GOx for tumor killing. In addition, apoptosis assays further evidenced that mannose and GOx can induce significant cell death ( Fig. 1D and 1E). Collectively, these results confirmed our proposal that mannose plus GOx can induce cancer cell death in a synergistic manner (supplementary Fig. S1).

**Figure 1:**
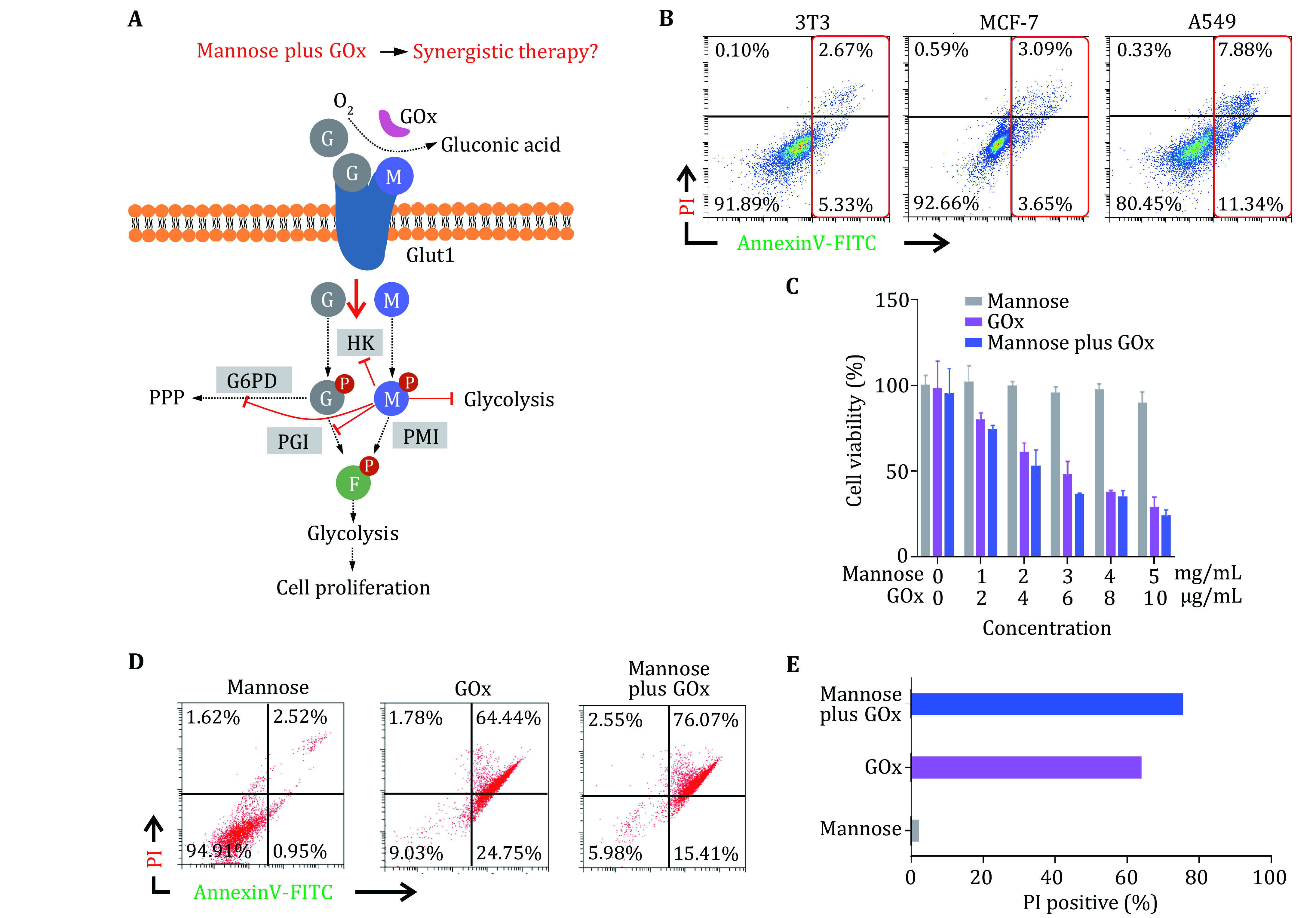
Evaluation of the synergistic therapeutic efficacy of free mannose plus GOx *in vitro*. **A** Schematic of glycolysis inhibition mechanism of free mannose plus GOx. G: glucose; M: mannose; HK: hexokinases; G6PD: glucose-6-phosphate dehydrogenase; PPP: pentose phosphate pathway; PGI: phosphoglucose isomerase; PMI: phosphomannose isomerase. **B** Cell apoptosis analysis of 3T3, MCF-7 and A549 cells treated with 5 mg/mL mannose. **C**, **D** Cell viability ( **C**) and cell apoptosis analysis ( **D**) of A549 treated with various concentrations of mannose, GOx and mannose plus GOx. **E** Statistical data of PI-positive cells according to Panel D

### Fabrication of the nanospoiler

**Figure 2:**
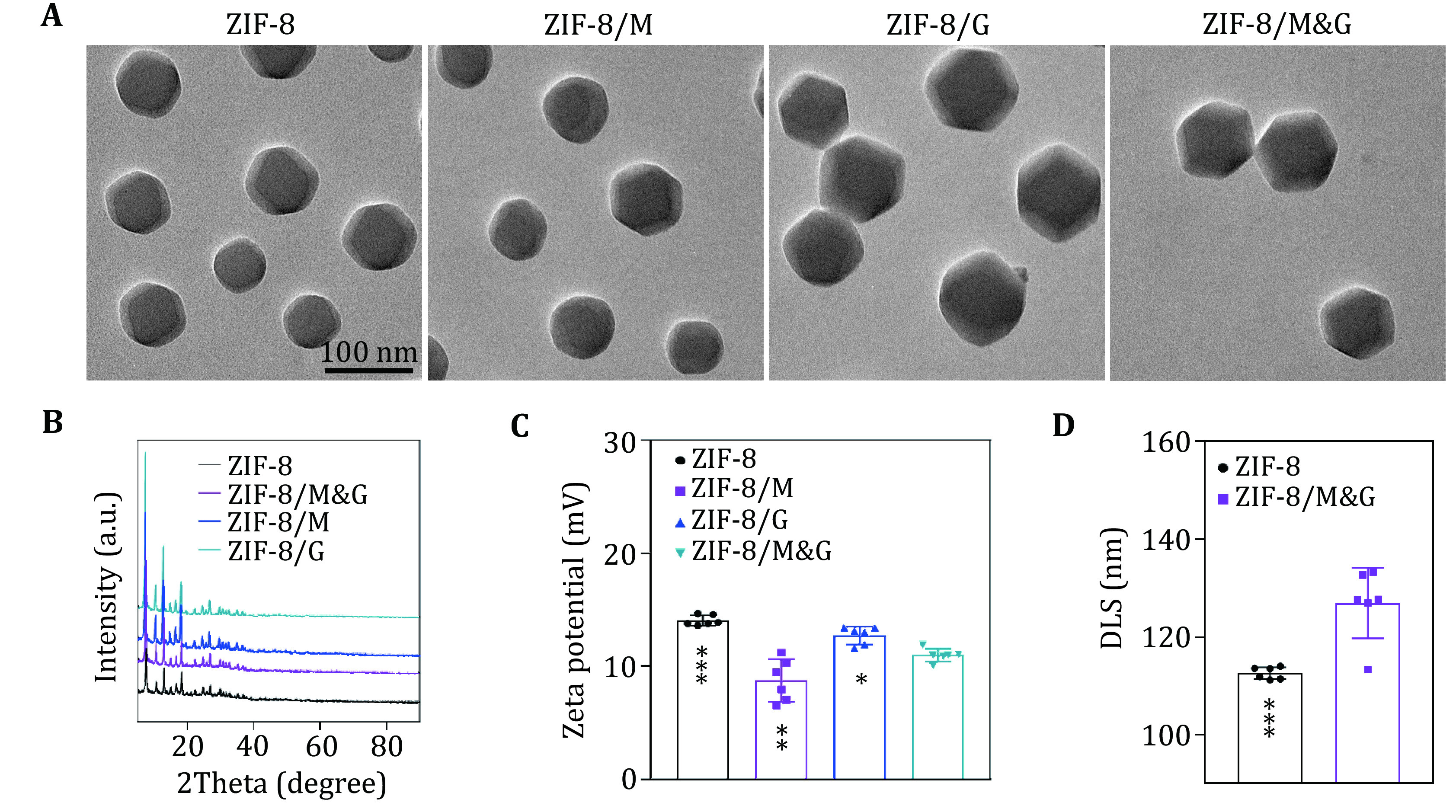
Characterization of the nanospoiler. **A** TEM images of ZIF-8, ZIF-8/M, ZIF-8/G and ZIF-8/M&G. **B** XRD patterns of ZIF-8, ZIF-8/M, ZIF-8/G and ZIF-8/M&G. **C** Zeta potential of ZIF-8, ZIF-8/M, ZIF-8/G and ZIF-8/M&G. **D** Comparation of DLS size of ZIF-8 and ZIF-8/M&G. *n* = 5, * *P* < 0.05, ** *P* < 0.01, **** *P* < 0.0001 and ns: not significant ( *P* > 0.05). Data represent mean ± SD ( **C**, **D**)

Free therapeutics such as chemical drugs and biological agents exhibit poor stability and tumor accumulation efficiency. Nanomaterials-based drug delivery systems have been wildly applied to overcome the above difficulties for therapeutic delivery. For example, zeolitic imidazolate frameworks (ZIF-8) hold properties of pH-responsive degradation, high compatibility, and easy preparation, which draw significant attention in drug delivery and tumor therapy (Gao *et al.* 2019). Thus, in this work, we chose the ZIF-8 nanoparticles as the nanocarrier for the simultaneous delivery of mannose and GOx. As shown in Fig. 2A, ZIF-8 nanoparticles displayed a regular morphology. After loading mannose (ZIF-8/M), GOx (ZIF-8/G), or mannose and GOX (ZIF-8/M&G), the particles still hold integrated morphology and dispersity. Powder X-ray diffraction (PXRD) studies showed that the ZIF-8 maintained its structure after loading mannose, GOx, or mannose and GOX ( Fig. 2B). Zeta potential analysis demonstrated the ZIF-8 presented positive charges when encapsulating with different cargos ( Fig. 2C and supplementary Fig. S2). As illustrated in Fig. 2D and supplementary Fig. S3, the nanospoliler, ZIF-8/M&G, had a larger size (125 nm) than primary ZIF-8 in water, which was acquired by dynamic light scattering (DLS) measurement. The low polydispersity index (PDI) further confirmed the excellent dispersity of ZIF-8/M&G.

### *In vitro* experiments

**Figure 3:**
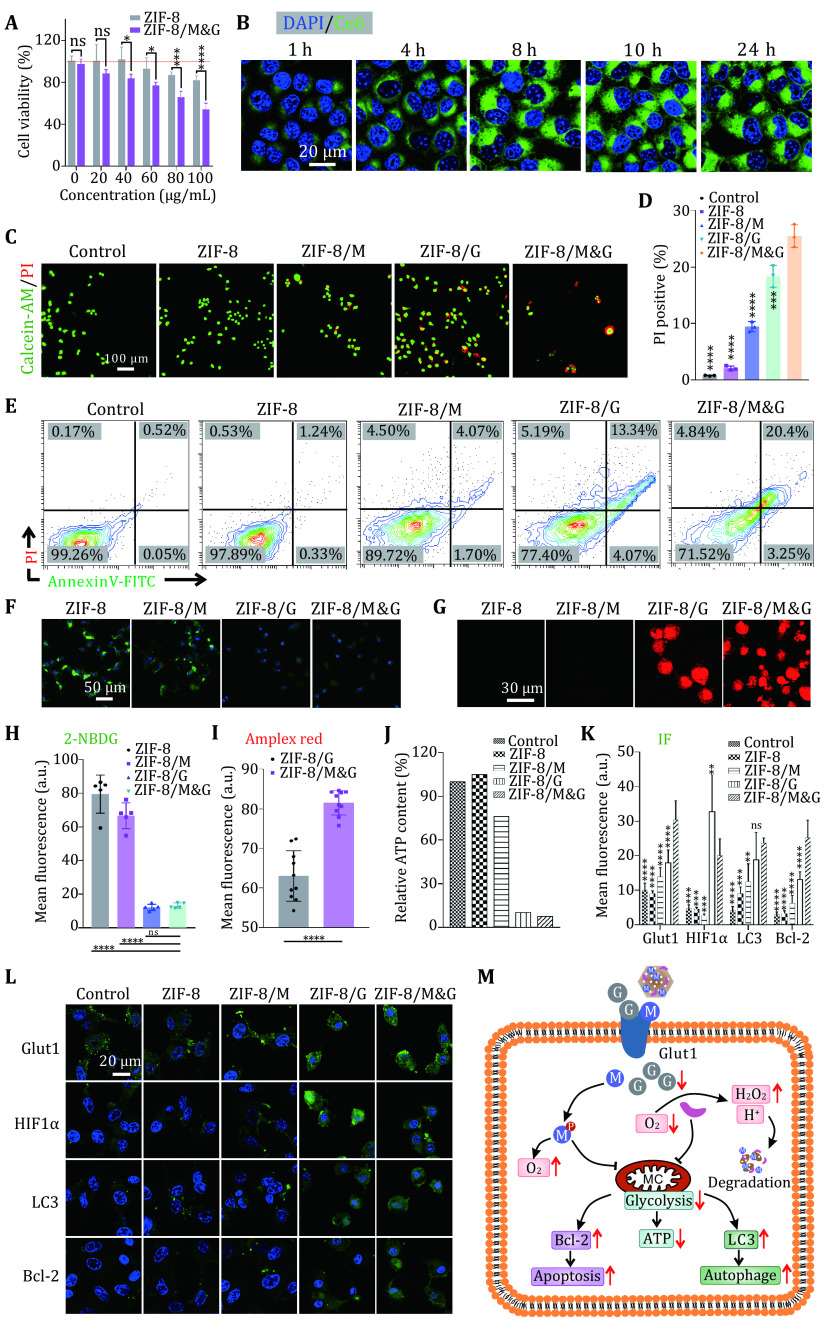


Next, we studied *in vitro* therapeutic efficiency of the ZIF-8/M&G. As shown in Fig. 3A, ZIF-8 had a minimal effect on A549 cell viability. By contrast, ZIF-8/M&G exhibited high cytotoxicity with concentration increasing. This result implied the therapeutic ability of the as-prepared ZIF-8/M&G. Thereafter, we studied the therapeutic mechanism of ZIF-8/M&G for A549 cells. Previous studies showed ZIF-8 could enter into cells via an endocytic pathway (Poddar *et al*. 2019). We first evaluated the drug delivery ability of ZIF-8 nanoparticles and fluorescent photosensitizer chlorin e6 (Ce6) as the surrogate of mannose and GOx. As can be seen from Fig. 3B and supplementary Fig. S4, the green fluorescent signals increased with incubation time and reached saturation at eight hours, showing that ZIF-8 has the ability to deliver therapeutic in cells. Additionally, A549 cells showed a distinct uptake quantity compared with healthy cells in a short time (2 h). Then, the live/dead staining assay further confirmed the excellent synergistic efficacy of ZIF-8/M&G on A549. Additionally, A549 cell treated with various formulations and cellular apoptosis was studied. As shown in Fig. 3D and 3E, ZIF-8 almost had no toxicity on cells compared to the control while cells treated with ZIF-8/M exhibited minimal apoptosis. By contrast, ZIF-8/G caused a high apoptosis rate, which arose from glucose consumption and related glycolysis disturbance by GOx. Significantly, ZIF-8/M&G gave rise to more cellular apoptosis than ZIF-8/M and ZIF-8/G, demonstrating that our designed nanospoiler has the potential for tumor treatment through synergistic glycolysis disturbance.

**Figure 3:** *In vitro* synergistic therapy and mechanism of ZIF-8/M&G. **A** Cell viability of A549 cells treated with ZIF-8 and ZIF-8/M&G with various concentrations. **B** Fluorescent images of A549 incubated with ZIF-8/Ce6 for different time points. **C** Live/dead cell fluorescent staining of A549 after treating with different formulas. **D**, **E** PI positive cells ( **D**) and cell apoptosis analysis ( **E**) of A549 treated with various formulas. **F**– **I** Fluorescent images and statistics results of intracellular glucose ( **F**, **H**) and H _2_O _2_ ( **G**, **I**) level. **J** Intracellular ATP content of A549 treated with different formulas. **K**, **L** Immunofluorescence images ( **L**) and statistical results ( **K**) of A549 treated with different formulas. **M** Schematic represents the mechanism of synergistic therapy of ZIF-8/M&G. ZIF-8/M&G nanoparticles induce expression of LC3 and Bcl-2, thus inducing cell death via autophagy and apoptosis simultaneously. Scale bars are shown as indicated. *n* ≥ 3, * *P* < 0.05, ** *P* < 0.01, **** *P* < 0.0001 and ns: not significant ( *P* > 0.05). Data represent mean ± SD ( **A**, **D**, **H**, **I** and **K**)

Subsequently, we further studied metabolic changes of tumor cells upon treatment with the nanospoiler. Firstly, 2-NBDG, fluorescent glucose, was used to monitor glucose uptake. As depicted in Fig. 3F and 3H, 2-NBDG signals decreased after incubating with ZIF-8/M compared to the ZIF-8 treated cells. And there were almost no fluorescent signals in cells treated with ZIF-8/G and ZIF-8/M&G particles. Those results implied that ZIF-8/M&G can attenuate glucose uptake, which may hinge on reduced cellular viability. GOx catalysis glucose and oxygen into gluconic acid and H _2_O _2_. Thus, glucose catalysis was next investigated by detection of the H _2_O _2_ content. According to fluorescent imaging ( Fig. 3G), both ZIF-8/G and ZIF-8/M&G treated cells have a high concentration of H _2_O _2_, suggesting that ZIF-8/G and ZIF-8/M&G hold the ability for glucose consumption. And ZIF-8/M&G had a higher fluorescent intensity than ZIF-8/G ( Fig. 3I), demonstrating that mannose can enhance the catalysis of GOx for glucose. Adenosine triphosphate (ATP) generated from glycolysis is the energy source of cells. Decidedly, disturbance of glycolysis is able to inhibit the generation of ATP in cells (Akram 2013). As can be seen from Fig. 3G, ZIF-8/M can reduce ATP generation, which further confirmed the ability of mannose for glycolysis disturbance. In addition, the high ATP suppression of ZIF-8/G contributed to the excellent catalytic capacity of GOx for glucose. Unsurprisingly, ZIF-8/M&G inhibited about 90% of ATP generation. This provides further evidence that mannose plus GOx is able to compromise ATP production and glycolysis disturbance.

Furthermore, several proteins related to glucose metabolism were investigated through immunofluorescence staining. As illustrated in Fig. 3K and 3L, both ZIF-8/M and ZIF-8/G upregulated the glut1 (a glucose transporter). And the ZIF-8/M&G triggered higher glut1 expression than ZIF-8/M and ZIF-8/G. These results are consistent with the idea that glucose consumption will lead to overexpression of glucose transporter. This may be the reason that mannose can increase the glucose uptake of cells. Immunofluorescent staining of HIF1α presented that ZIF-8/M alleviated the hypoxia of cells, which may result from mannose affecting glucose metabolism. The high HIF1α signals in ZIF-8/G-treated cells exhibited oxygen consumption due to the catalysis of glucose by GOx. However, ZIF-8/M&G relieved the hypoxia of cells compared to ZIF-8/G, which may result from the disturbance of glucose metabolism and thus will enhance the catalytic activity of GOx. In addition, high expression of LC3 and Bcl-2 proteins implied that ZIF-8/M&G stimulated cellular autophagy and apoptosis, respectively. All considered, mannose synergistic with GOx can induce cell death by ATP inhibition, autophagy activation, and the upgradation of apoptosis protein.

### Biosafety evaluation

Before the *in vivo* study, we evaluated the biosafety of the fabricated particles. According to the hemolysis assay, both ZIF-8 and ZIF-8/M&G only had about 3% hemolysis rate even at 15 mg/mL ( Fig. 4A). In addition, ZIF-8/M&G was intravenously injected in mice at a dosage of 16 mg/kg and PBS as the control. Two months late, mice were sacrificed and their hematology and biochemistry as well as tissue histology were analyzed. As shown in Figs. 4B, 4C and supplementary Fig. S5, there had no obvious differences in ZIF-8/M&G and PBS-injected mice, demonstrating that the ZIF-8/M&G possessed excellent long-term biocompatibility.

**Figure 4:** Evaluation of biosafety of ZIF-8/M&G. **A** Hemolysis ratio of ZIF-8/M&G nanoparticles. Negative (N), PBS; Positive (P), 0.5% Triton-X. **B**, **C** H&E staining of major organs ( **B**) and blood biochemical parameters ( **C**) of mice after receiving ZIF-8/M&G nanoparticles at a dosage of 16 mg/kg for two months, *n* = 5. Scale bars are 100 μm. *n* = 5, * *P* < 0.05, ** *P* < 0.01, **** *P* < 0.0001 and ns: not significant ( *P* > 0.05). Data represent mean ± SD ( **C**)

### Biodistribution analyse

After demonstrating the *in vitro* synergistic therapeutic function and biosafety of ZIF-8/M&G, we thus intend to test the biodistribution of the ZIF-8/M&G in the tumor-bearing tumor model. We firstly loaded fluorescent ICG (indocyanine green) molecules as the delegator of M&G in ZIF-8 nanoparticles (hereafter referred to as ZIF-8/ICG). Then, we used *in vivo* fluorescence imaging to track the ZIF-8/ICG in 4T1 tumor-bearing mice and the free ICG as a control. As shown in Fig. 5A, ZIF-8/ICG mainly accumulated at the tumor site after intravenous injection at 12 h. And ZIF-8/ICG still has a higher fluorescence intensity compared to the free ICG after 72 h injection ( Fig. 5A and 5B), indicating the abilities of efficient tumor accumulation and retention of the therapeutic-loaded ZIF-8 nanoparticles. The *ex vivo* images of the tumor and major organs collected from 72 h post-injection further implied the excellent tumor accumulation ability of ZIF-8/ICG ( Fig. 5C and 5D).

**Figure 5:** Evaluation of the biodistribution of ZIF-8/ICG nanoparticles. **A** *In vivo* fluorescence images of tumor-bearing Balb/c mice after intravenous injection of ZIF-8/ICG at different time points. **B** The fluorescence intensity of tumors at different time points. **C**, **D** The fluorescence images ( **C**) and intensity ( **D**) of tumors and major organs at 72 h time point. *n* = 4, * *P* < 0.05, ** *P* < 0.01, **** *P* < 0.0001 and ns: not significant ( *P* > 0.05). Data represent mean ± SD ( **B**, **D**)

### *In vivo* synergistic tumor therapy

Encouraged by its excellent therapeutic efficacy *in vitro* and biocompatibility, we next evaluated the *in vivo* tumor therapeutic efficiency of ZIF-8/M&G in the A549 tumor-bearing murine model. As shown in Fig. 6A, tumor size in group ZIF-8 grew rapidly while groups ZIF-8/M and ZIF-8/G delayed the growth of tumors. Specifically, the synergistic glycolysis disturb group (ZIF-8/M&G) could eradicate the tumors. The tumor weights ( Fig. 6B) and photographs ( Fig. 6C) of mice after treatments further illustrated the significant therapeutic efficacy of ZIF-8/M&G. Figure 6D represented the changes in murine weights after treatment with various formulas. The images of H&E histologic staining and Ki67 immunohistochemical staining showed that group ZIF-8/M&G induced more cell death and inhibited more cellular proliferation ( Fig. 6E). The terminal deoxynucleotidyl transferase dUTP nick end labeling (TUNEL) indicated more cellular apoptosis in group ZIF-8/M&G ( Fig. 6F). These results confirmed that synergistic glycolysis disturbance by mannose plus GOx had a better performance in tumor treatment than mannose or GOx was exercised individually. Additionally, our prepared nanoplatform did not have a negligible influence on the health of the mice after administering the treatments, as indicated by pathological staining of the major tissues ( Fig. 6G).

**Figure 6:** *In vivo* tumor therapy by ZIF-8/M&G. **A** Tumor growth curves of mice with different treatments ( *n* = 5). **B**, **C** Tumor weights ( **B**) and tumor photos ( **C**) of mice after treating with various formulas, *n* = 5. **D** Average body weights of A549 tumor-bearing mice after various treatments. Error bars are based on the standard error of the mean ( *n* = 5). **E** Representative H&E and Ki67 staining images of tumor slices at 24 h after treatment. Scale bars represent 100 µm. **F** TUNEL staining of tumor slices at 24 h posttreatment; the scale bars represent 100 µm. **G** H&E staining images of tissue sections from the heart, liver, spleen, lung, kidney, and intestine of tumor-bearing Balb/c mice receiving various treatments. Scale bars represent 100 µm. *n* = 5, * *P* < 0.05, ** *P* < 0.01, **** *P* < 0.0001 and ns: not significant ( *P* > 0.05). Data represent mean ± SD ( **A**, **B**)

## CONCLUSION

In summary, for the first time, we fabricated a nanospoliler based on ZIF-8 encapsulated mannose and GOx for tumor treatment by synergistic glycolysis disturbance. This highly biocompatible nanospoliler not only accumulated in tumor sites via the enhanced permeability and retention effect but also exhibited functions of mannose and GOx. We found that mannose can enhance the activity of GOx by alleviating hypoxia of the tumor microenvironment. In addition, we also found that the significant tumor therapeutic efficacy of nanospoliler arose from its abilities of ATP inhibition, autophagy activation, and the upgradation of apoptosis protein. This study provided new insights into the application of concerted glycolysis disturbance for high-efficiency tumor therapy, which may extend to other glycolysis modulators for the tumor or other disease treatments.

## MATERIALS AND METHODS

### Materials

Zn(NO _3_) _2_·6H _2_O, 2-methylimidazole (2-mIm), mannose, Calcein-AM, propidium iodide (PI), and chlorin e6 (Ce6) were bought from Shanghai Aladdin Biochemical Technology Co., Ltd. Gluconic oxidase (GOx) was got from Beijing Solarbio Science & Technology Co., Ltd. CCK-8 and annexin V-FITC/PI apoptosis kits were acquired from Dojindo. 2-NBDG was obtained from Amgicam. 4',6-diamidino-2-phenylindole (DAPI), and amplex red were purchased from Beyotime. All cell cultured media including Dulbecco’s modified Eagle medium (DMEM), phosphate buffer solution (PBS), and fetal bovine serum (FBS) were received from Hyclone. Ultrapure water (18.2 MΩ; Millipore Co.) was used in this study.

### Synthesis of ZIF-8/M&G

The ZIF-8/M&G nanoparticle was synthesized through one-pot biomineralization methods. Briefly, 15 mg Zn(NO _3_) _2_·6H _2_O, 5 mg mannose, and 40 μg GOx were dissolved in 0.5 mL methyl alcohol to get Solution A, and 2-mIm (33 mg) was dissolved in 1 mL ultrapure water to get Solution B. Then, Solution A was added to Solution B dropwise under vigorous stirring for 5 min. After that, the mixture was washed several times to get ZIF-8/M&G. By using the same methods, ZIF-8, ZIF-8/M and ZIF-8/G nanoparticles were obtained.

### Characterization of ZIF-8/M&G

The morphology of synthesized ZIF-8, ZIF-8/M, ZIF-8/G and ZIF-8/M&G was imaged by transmission electron microscopy (Hitachi HT7700). The hydrodynamic diameter and zeta potential of nanoparticles were measured on a Malvern Zetasizer (ZEN3600, Malvern Instruments). The crystal structure was explored by an X-ray diffraction apparatus (X’Pert PRO PANalytical B.V.). The *in vivo* biodistribution of fluorescence images was recorded by an imaging instrument (IVIS) spectrum system (PerkinElmer).

### Cell culture

A549 cells and HUVEC cells were cultured in DMEM media supplemented with 10% FBS and 1% streptomycin/penicillin and incubated in a cell incubator (37 °C, 5% CO _2_).

### Cell viabilities of ZIF-8/M&G

The CCK-8 assay was conducted to evaluate the cell viability of ZIF-8/M&G to A549 cells. Cells were seeded in 96-well plates at a density of 1 × 10 ^4^ per well overnight. Then different concentrations of free mannose, free GOx, mannose plus GOx, ZIF-8 and ZIF-8/M&G were added to wells and incubated for another 24 h. After that, the cell media was replaced with fresh media and added 10 μL CCK-8 to each well for another 2 h incubation. Finally, the microplate reader (Thermo MultiscanGO) was applied to measure the absorption at 450 nm to evaluate the cell viability.

### Cellular uptake behaviour of ZIF-8

The ZIF-8 loaded with Ce6 (a type of photosensitizer) (ZIF-8/Ce6) and ZIF-8 loaded with Dil (a fluorescent dye) (ZIF-8/Dil) were synthesized by a one-pot method. A549 cells were seeded in small confocal dishes at a density of 1 × 10 ^5^ per dish overnight. Then 80 μg/mL ZIF-8/Ce6 was added to dishes and incubated at different time points (1, 4, 8, 12, and 24 h). Cell samples were immobilized with 4% PFA for 10 min and stained with DAPI for 10 min. The fluorescent intensity of samples was imaged by a CLSM (Zeiss, German). The statistics results were obtained by Image J software. To study the uptake behaviour of ZIF-8 nanoparticles by healthy cells and tumor cells, A549 and HUVEC cells were seeded in small confocal dishes and incubated with 80 μg/mL ZIF-8/Dil for 2 h. The fluorescent intensity of samples was imaged by a CLSM.

### Live/dead staining assay

80 μg/mL of ZIF-8, ZIF-8/M, ZIF-8/G and ZIF-8/MG was added to cell samples in confocal dishes and incubated for 24 h. Subsequently, the Calcein-AM/PI staining kit was used to stain cell samples and fluorescence was imaged using CLSM.

### Apoptosis assay

Cells were seeded in 6-well plates overnight at a density of 1.2 × 10 ^6^ per well. Then, 80 μg/mL of ZIF-8, ZIF-8/M, ZIF-8/G and ZIF-8/MG were added to wells and incubated for 24 h. Finally, the annexin V-FITC/PI apoptosis kit was utilized to stain cell samples and fluorescence was measured by a flow cytometer (NanoCyte, Biosciences).

### Cellular ATP detection

80 μg/mL of ZIF-8, ZIF-8/M, ZIF-8/G and ZIF-8/MG were added to cell samples in 6-well plates and incubated for 24 h. Then, cell samples were lysed and intracellular ATP content was detected by an ATP detection kit.

### Glucose uptake study

80 μg/mL of ZIF-8, ZIF-8/M, ZIF-8/G and ZIF-8/MG were added to cell samples cultured in confocal dishes and incubated for 24 h. After that, the fresh media containing 50 μg/mL 2-NBDG was replaced for another 30 min incubation. Finally, the fluorescence of 2-NBDG was imaged by CLSM.

### Cellular H _2_O _2_ detection

80 μg/mL of ZIF-8, ZIF-8/M, ZIF-8/G and ZIF-8/MG were added to cell samples in confocal dishes and incubated for 24 h. After that, the fresh media containing 5 μg/mL amplex red and 1 U/mL HRP was replaced for another 10 min incubation. Finally, the fluorescence of amplex red dye was imaged in a CLSM.

### Immunofluorescence assay

80 μg/mL of ZIF-8, ZIF-8/M, ZIF-8/G and ZIF-8/MG was added to cell samples in confocal dishes and incubated for 24 h. After that, cell samples were immobilized with 4% PFA for 30 min and permeabilized with 0.1% Triton X-100 for 15 min. Next, the blocking step was carried out by PBS supplemented with 1% BSA for 30 min. Then, the samples were incubated with anti-GLUT1 (1∶100, E-AB-31556, Elabscience), anti-Bcl-2 (1∶100, E-AB-60012, Elabscience), anti-HIF1α (1∶100, E-AB-31662, Elabscience), anti-LC3A/3B (1∶100, E-AB-61027, Elabscience) for 24 h, followed by labelling with secondary antibodies at room temperature for 1 h. Finally, cell samples were stained with DAPI and imaged by CLSM.

### Hemolysis test

The hemolysis analysis was conducted according to our previous report. Mouse blood was centrifuged several times to obtain red blood cells. Then an equal volume of PBS was added. After that, various concentrations of ZIF-8 and ZIF-8/M&G nanoparticles were added to 100 μL red blood cells and incubated at 37 °C for 2 h. The samples were centrifuged and the supernatant was collected and the absorption at 576 nm was read to measure the hemolytic rate of nanoparticles.

### *In vivo* biosafety assay

Male Balb/c mice (four weeks) were purchased from Shanghai Slack Laboratory Animal Co., Ltd., and the experiments were implemented in accordance with protocols approved by the Animal Experimental Ethics Committee of Fujian Normal University. Ten mice were i.v. injected 16 mg/kg ZIF-8 or ZIF-8/M&G on Day 1, Day 5, and Day 10. After two months, mice were sacrificed and blood and serum were collected for hematology and biochemistry analysis. In addition, the major organs were collected, sliced, and stained for H&E analysis.

### *In vivo* synergistic starvation therapy

Twenty tumor-bearing mice were divided into four groups: ZIF-8, ZIF-8/M, ZIF-8/G and ZIF-8/M&G (the nanoparticles were i.v. injected into mice at a dosage of 16 mg/kg on Day 1, Day 5, and Day 10). The body weights and tumor weights of mice were recorded every two days. On Day 17, mice were sacrificed and major organs were collected for H&E staining. To further evaluate synergistic therapeutic efficacy, mice with A549 tumors were i.v. injected different nanoparticles and sacrificed on Day 5. The tumors of each group were collected and sliced for H&E staining, TUNEL staining, and Ki-67 staining.

### Statistical analysis

All data in this study were analyzed via GraphPad Prism and presented as mean ± SD. The unpaired *t*-test with Welch’s correction was used for the two-group comparisons. The one-way ANOVA was used for more than two-group comparisons. *P*-values of 0.05 or less were considered statistically significant.

## Conflict of interest

Xuemei Zeng, Yihang Ruan, Lun Wang, Jinpeng Deng and Shuangqian Yan declare that they have no conflict of interest.
